# A Single Dose of Piperacillin Plus Tazobactam Gel as an Adjunct to Professional Mechanical Plaque Removal (PMPR) in Patients with Peri-Implant Mucositis: A 6-Month Double-Blind Randomized Clinical Trial

**DOI:** 10.3390/antibiotics13030269

**Published:** 2024-03-17

**Authors:** Ioana Ilyes, Marius Boariu, Darian Rusu, Vincenzo Iorio-Siciliano, Octavia Vela, Simina Boia, Georgios Kardaras, Petra Șurlin, Horia Calniceanu, Holger Jentsch, Alexandru Lodin, Stefan-Ioan Stratul

**Affiliations:** 1 Department of Periodontology, Faculty of Dental Medicine, Anton Sculean Research Center for Periodontal and Peri-Implant Diseases, “Victor Babes” University of Medicine and Pharmacy, 300041 Timisoara, Romania; ioana.veja@umft.ro (I.I.); rusu.darian@umft.ro (D.R.); vela.octavia@umft.ro (O.V.); simina.boia@umft.ro (S.B.); kardaras.georgios@umft.ro (G.K.); calniceanu.horia@umft.ro (H.C.); stratul.stefan@umft.ro (S.-I.S.); 2 Department of Endodontics, Faculty of Dental Medicine, TADERP Research Center, “Victor Babes” University of Medicine and Pharmacy, 300041 Timisoara, Romania; 3 Department of Periodontology, University of Naples Federico II, 80131 Naples, Italy; enzois@libero.it; 4 Department of Periodontology, Faculty of Dental Medicine, University of Medicine and Pharmacy, 200349 Craiova, Romania; petra.surlin@umfcv.ro; 5 Medical Faculty, University of Leipzig, 04103 Leipzig, Germany; jenh@medizin.uni-leipzig.de; 6 Department Basis of Electronics, Faculty of Electronics, Telecommunications and Information Technology, Technical University of Cluj-Napoca, 400114 Cluj-Napoca, Romania; alexandru.lodin@bel.utcluj.ro

**Keywords:** peri-implant mucositis, clinical trial, local antibiotics, adjuvant therapy

## Abstract

Objectives: This randomized, placebo-controlled, double-masked clinical trial aimed to evaluate the clinical and microbiological efficacy of professional mechanical plaque removal (PMPR) with or without adjunctive application of piperacillin plus tazobactam gel in the treatment of peri-implant mucositis (PiM) for up to 6 months. Materials and Methods: The study included 31 patients with peri-implant mucositis (bleeding on probing (BoP) > 1 at at least one site at baseline, absence of peri-implant bone loss compared with a previous radiograph). After randomized assignment to test and control groups, patients received full-mouth supragingival scaling with or without piperacillin plus tazobactam gel. Clinical examination was performed at baseline and after 3 and 6 months, and a microbiological examination was performed at baseline and after 3 months. Results: After six months, both treatment modalities resulted in significant reductions and improvements in clinical parameters at the implant sites. Neither study group achieved a complete resolution of PiM (i.e., BoP ≤ 1 per implant). The number of implants with BoP decreased statistically significantly between subsequent time points (*p* < 0.001) in both the test and the control group. Significant BoP differences (*p* = 0.039) were observed between groups at 6 months (difference to baseline) following therapy. Conclusions: Within the limitations of the present study, the single use of a slow-release, locally applied antibiotic combination of piperacillin and tazobactam gel, adjunctive to PMPR, showed an improvement in clinical variable of implants diagnosed with PiM. The adjunctive treatment resulted in higher BoP reduction when compared to the control, but no significant differences were observed regarding the changes in other clinical and microbiological parameters.

## 1. Introduction

Peri-implant mucositis (PiM) was recently defined as a pathological condition that frequently occurs around dental implants and is characterized by inflammation confined to the peri-implant soft tissue mucosa without any bone loss [1]. The detailed clinically diagnostic definition of PiM is based on the following criteria: (a) signs of inflammation around the implant (red instead of pink, swollen tissues instead of no swelling, soft tissues instead of firm ones); (b) heavy bleeding and/or pus on probing; (c) deeper probing than before; and (d) no more bone loss after the initial remodeling [2]. PiM occurs in about half of the population with dental implants [2,3,4], and untreated PiM is the precursor to peri-implantitis (PI), with a prevalence estimate of 43% [5,6]. If appropriate measures are used to control the biofilm, PiM is a reversible condition [7,8]. The definition of PiM has been updated as a result of the Implant Dentistry Core Outcome Set and Measurement (ID-COSM) initiative consensus modification [9] as follows: the absence of bone loss beyond the crestal bone level changes resulting from initial bone remodeling, the presence of bleeding (more than one spot at a location around the implant or the presence of a line of bleeding or profuse bleeding at any location), and/or suppuration on gentle probing (S3 level CPG, Herrera) [6]. Plaque buildup is the main cause of PiM [10,11], and the time it takes for mucositis to turn into PI is linked to function time, especially when supportive maintenance treatment is not available [12,13]. 

PiM prevention and treatment are becoming more important [14] because the condition can be treated with proper inflammation management; treating PiM is thought to be the best way to avoid PI [15]. An excellent preventive and therapeutic strategy must identify the critical elements linked to the genesis and risk factors of peri-implant disorders [16]. Major contributing factors include: (a) biofilm accumulation is associated with the presence of PiM; (b) compliance with supportive implant therapy (SPIC); (c) accessibility for biofilm removal around implant-supported prostheses; (d) dimensions of peri-implant keratinized mucosa; and (e) excess cement [1]. Additional etiologic general risk indicators affecting host susceptibility to biofilm-induced PiM are smoking, radiation therapy, and diabetes mellitus [1].

Therapy for PiM often involves providing oral hygiene guidance, as well as mechanical removal of the plaque and calculus [15]. Although there are clinical improvements, achieving total disease remission of PiM may only be possible in select cases [17,18]. Research shows that using mechanical debridement by itself is not very effective, but additional methods could enhance the overall success of the treatment [19,20,21]. Local or systemic antibiotics, antiseptics, lasers, air-abrasive devices, and/or photodynamic therapy are suggested as additional treatments to improve the effectiveness of non-surgical treatment for additional implant surface decontamination [18,20,21,22,23,24,25,26,27,28,29,30,31]. Chlorhexidine (CHX) is the most commonly utilized additional agent for controlling the biofilm around implants [32]. Antibiotics utilized in clinical trials complementing mechanical treatment in PiM included tetracycline fibers for ten days [33], slow-release doxycycline [34], and minocycline microspheres [35]. These approaches showed improvements in clinical parameters such as bleeding on probing (BoP) and probing depth (PD). Piperacillin/tazobactam is a blend of β-lactam and β-lactamase inhibitors that effectively eliminate a broad spectrum of bacteria, including numerous pathogens that produce β-lactamases. It is effective against a wide range of aerobic and anaerobic bacteria, including both Gram-positive and Gram-negative types [36]. This medication is beneficial for treating patients with polymicrobial infections caused by aerobic or anaerobic β-lactamase-producing bacteria. It is effective against moderate to severe infections in various parts of the body, such as the lower respiratory tract, urinary tract, skin, gynecologic area, bones, and joints, which can cause intra-abdominal infections and septicemia due to susceptible organisms [37]. Piperacillin is a semisynthetic penicillin, while tazobactam increases the effectiveness of piperacillin against strains of *S. aureus*, *H. influenzae*, *Bacteroides*, and other Gram-negative bacteria that produce β-lactamase [38]. Tazobactam extends the bactericidal action of piperacillin, increasing the range of bacteria that piperacillin can effectively target by inhibiting the activity of β-lactamase enzymes. It is a β-lactam antibiotic that acts by inhibiting numerous β-lactamases that often produce resistance to penicillin [39]. None of the microorganisms obtained from 16 individuals suffering from dental-related infections affecting various maxillofacial and neck regions, who also exhibited symptoms of sepsis in laboratory examinations, displayed any resistance to the combination of piperacillin and tazobactam. Early administration of this antibiotic combination is the recommended initial treatment for cellulitis originating from dental infections [40].

Recently, a novel formulation of piperacillin plus tazobactam was introduced in periodontal practice for local drug delivery (LDD). It is a patented mixture of piperacillin and tazobactam combined in a polymeric volatile carrier. The mixture leaves a pellicle on the dried, instrumented surface, which turns into a gel with slow release. The product has been used as an adjunctive to subgingival instrumentation during step 2 of the treatment of severe periodontitis, with inconclusive results [41,42,43,44]. To the best of our knowledge, the clinical effects of this agent in the non-surgical treatment of PiM have not been reported so far. Currently, only a prospective clinical case series, with a surgical approach combining implantoplasty and reconstructive therapy with locally delivered antibiotic in the treatment of PI [45], has reported positive results. Clinical and microbiological data on the application of this formulation for treating biofilm-associated peri-implant infections are absent. Thus, its potential clinical benefits have yet to be discovered. This study was conducted to evaluate the effectiveness of professional mechanical plaque removal (PMPR) either in conjunction with or without the addition of a piperacillin plus tazobactam gel in PiM over a period of 6 months. The trial was a randomized, placebo-controlled, and double-blinded clinical trial.

## 2. Materials and Methods

The study design was approved by the Committee for Research Ethics of the “Victor Babeș” University of Medicine and Pharmacy Timisoara (approval No. 57/2022). It conforms to the requirements of the Declaration of Helsinki, as adopted by the 18th World Medical Assembly in 1964 and subsequently revised. The study is registered in the ISRCTN-94266769 Registry of Clinical Trials and follows the guidelines described in the CONSORT 2010 statement on clinical trials. 

### 2.1. Study Design and Study Group Allocation

**Figure 1:**
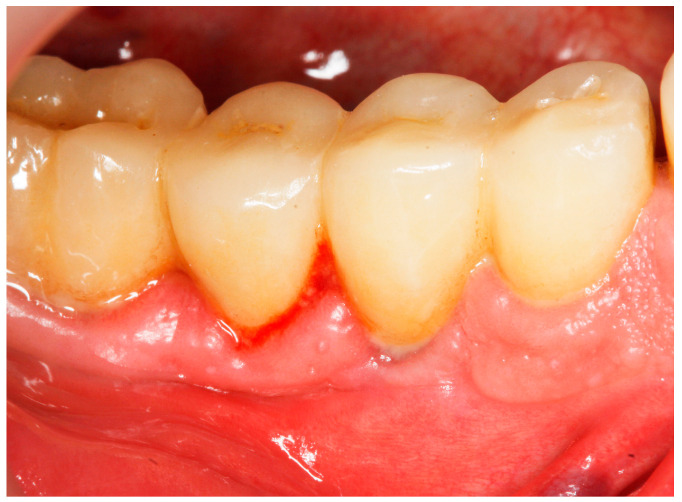
Pocket probing depth of 5 mm with BoP at baseline.

This study was designed as a prospective, double-blinded, randomized, placebo-controlled clinical trial with a parallel design and a 6-month duration. The study was carried out between January 2022 and September 2023. The study’s flow chart is reported in Figure 1, and data are reported according to the Consolidated Standards of Reporting (CONSORT) guidelines.

The data analyst recommended a randomization method using computer-generated random numbers with an allocation ratio of 1:1. Randomization was used to place patients into the test and control groups, and treatment assignment was kept secret by using opaque envelopes that had already been prepared, sealed, and labeled with the patient study number. These envelopes were opened by an external investigator (not involved in the outcome evaluations) directly after the PMPR. The two substances were blinded, so there were no differences between the two solutions and syringes. Consequently, neither the operator nor the patients were aware of the treatment administered. Blinding was achieved by blinding the patients, the examiner, and the operator.

### 2.2. Study Sample

Subjects were selected from patients of the Department of Periodontology of the “Victor Babeș” University of Medicine and Pharmacy Timișoara, Romania. The cohort of 34 patients was equally divided into one of the two groups: the test (piperacillin plus tazobactam) group or the control (placebo) group. The selection of patients who met the inclusion criteria was carried out until the desired number was achieved. One experienced investigator evaluated the subjects and was responsible for the patients’ enrollment process, according to the inclusion and exclusion criteria.

### 2.3. Inclusion Criteria

Subjects were included based on the following criteria:

(1) Age ≥ 18 years;
(2) Absence of relevant systemic disease;
(3) Partially edentulous patients with healthy or treated periodontal conditions enrolled in a regular supportive care program;
(4) Peri-implant mucositis defined as >1 implant site with presence of BoP and absence of radiographic bone loss compared with a previous radiograph [2];
(5) Implant in function for ≥1 year;
(6) Full-Mouth Plaque Score (FMPS) ≤ 25;
(7) Full-Mouth Bleeding Score (FMBS) ≤ 25.

### 2.4. Exclusion Criteria

Subjects were excluded based on the following criteria:

(1) Uncontrolled medical conditions;
(2) Pregnant or lactating females;
(3) Tobacco smoking ≥ 10 cigarettes/day;
(4) Untreated periodontal conditions;
(5) Removable implant—retained prosthesis;
(6) Use of antibiotics in the past 3 months;
(7) Subjects who received treatment for at least 2 weeks with any medication recognized to impact soft tissue conditions within 1 month of the baseline test;
(8) Other chronic systemic medications that could interfere with the study’s outcomes;
(9) Refusal to sign written informed consent.

The patients fulfilling the described criteria were invited to participate in the study, and all subjects were informed about the nature and purpose of the study. Each subject signed an informed consent document giving permission for the dental procedures and sampling of biological material. Oral information and written informed consent were provided before the commencement of the investigation for all participants. 

### 2.5. Hypothesis

The null hypothesis (H0) was that no statistically significant differences would be observed with respect to the clinical parameters of bleeding on probing (BoP), probing pocket depth (PPD), modified plaque index (mPlI), and modified bleeding index (mBI) between the two treatment modalities (i.e., adjunctive delivery of piperacillin plus tazobactam vs. placebo).

### 2.6. Clinical Examination

One blinded and calibrated investigator recorded the following clinical outcomes at 6 sites (three—mesial, central, and distal on the buccal and on the lingual/palatal side) by means of a manual periodontal probe (Click-Probe^®^ KerrHawe SA, CH-6934 Bioggio, Switzerland), applying a light probing force of approximately (0.20–0.25 N) around the selected implant (one per patient) at baseline and at 3 and 6 months of utilizing the supportive peri-implant care (SPIC) protocol [6]. Prior to the study, the examiner (specialist of Periodontology) was calibrated. The intra-examiner calibration for reliability testing resulted in κ = 0.92 for repeated measurements of PPD in two quadrants of five patients, other than the patients recruited for the study (to complete the evaluations needed for this study in a reliable and accurate manner that was consistent with current standards for clinical periodontal studies).

**At the implant level:**

(1) Implant probing pocket depth (PPD) was measured from the mucosal margin to the bottom of the probable pocket and evaluated at six sites per implant (i.e., disto-buccal, mid-buccal, mesio-buccal, mesio-lingual/palatal, mid-lingual/palatal, disto-lingual/palatal);
(2) Bleeding on probing (BoP) was recorded as 0 (no bleeding) or 1 (bleeding) after probing for PPD [15] (presence/absence of bleeding within 30 s following probing) (Figure 1);
(3) Suppuration on probing (SoP) was assessed according to either presence or absence of suppuration after probing;
(4) Modified Plaque Index (mPlI) was recorded as 0, 1, 2, 3 (Mombelli et al., 1987) [46];
(5) Modified Bleeding Index (mBI) was recorded as 0, 1, 2, 3 (Mombelli et al., 1987) [46].

**At the full-mouth level:**

(1) Full-mouth bleeding score (FMBS) represented the percentage of sites with bleeding on probing in the entire dentition (O’Leary, 1972) [47].
(2) Full-mouth plaque score (FMPS) represented the percentage of sites covered with plaque in the entire dentition (Claffey 1990) [48].

**Figure 2:**
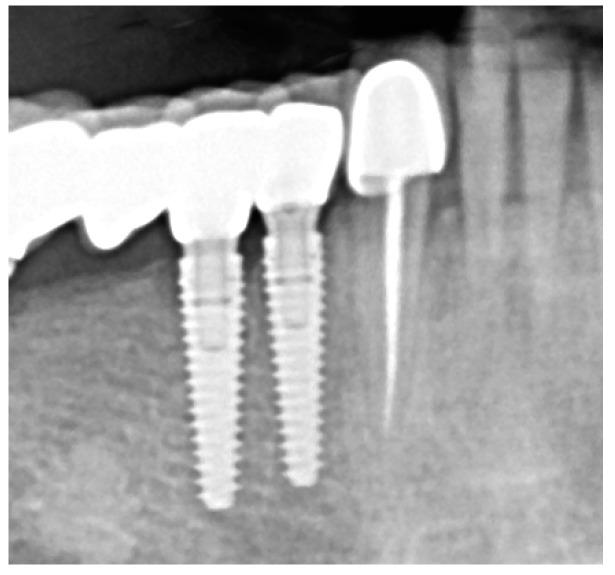
Implant without radiographic signs of crestal bone loss.

If the patient fulfilled the inclusion criteria, he or she received an informed agreement that he or she had 7 days to analyze, and he or she had to sign it to be included in the study. Peri-implant bone levels were measured using digitally scanned intraoral radiographs (Figure 2). The data were recorded in the periodontal sheet of the University of Bern, saved in PDF-format, printed, and included in the observation file of each patient.

### 2.7. Microbiological Examination

Microbiological subgingival samples were obtained from the deepest site of the included implant per implant in each patient (from the deepest of the 6 exanimated sites per selected implant). This site was used as the reference site for the samples collected at baseline and three months. Gingival crevicular fluids were obtained for microbiological evaluation as follows: The site was isolated with cotton rolls, the overgrowth plaque was removed with a curette sterile cotton pellet, the gingival surface was dried, and the paper cones were gently inserted into the site; contamination with blood or saliva was avoided [49]. The samples were obtained by inserting two sterile ISO #30 paper cones (ProTaper, Dentsply Sirona, Chemin du Verger, Switzerland) into the site, which were left in place for 30 s for saturation [50]. Samples were obtained at baseline (prior to the patient’s treatment) and three months after the initial evaluation. Detection of bacteria associated with periodontitis, i.e., *Aggregatibacter actinomycetemcomitans* (*A. actinomycetemcomitans*), *Porphyromonas gingivalis* (*P. gingivalis*), *Prevotella intermedia* (*P. intermedia*), *Tannerella forsythia* (*T. forsythia*), and *Treponema denticola* (*T. denticola*), was carried out via molecular genetic analysis of the samples taken. The presence of these bacteria was assessed using a commercial kit, micro-IDent^®^ (Hain Lifescience, Nehren, Germany). The same sites were used to collect the microbiological samples during the 3-month reevaluation period. 

### 2.8. Treatment Procedures

The SPIC specific protocol included (at baseline and at 3 and 6 months):

- Medical history update, risk assessment (interview);
- OH behavior improvements;
- Reinforcing of risk factor control (e.g., smoking, glycemic control);
- PMPR, individualized OH recommendations, for entire dentition/implants;
- Recall interval of 3 months [6].

**Figure 3:**
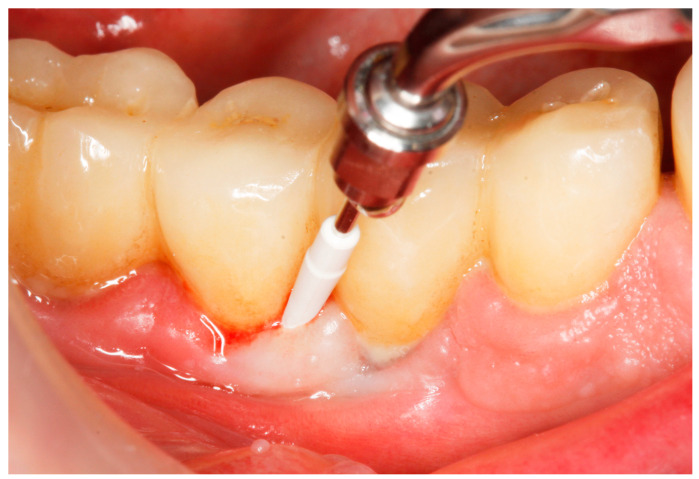
Mechanical debridement by means of an ultrasonic scaler with a plastic tip.

**Figure 4:**
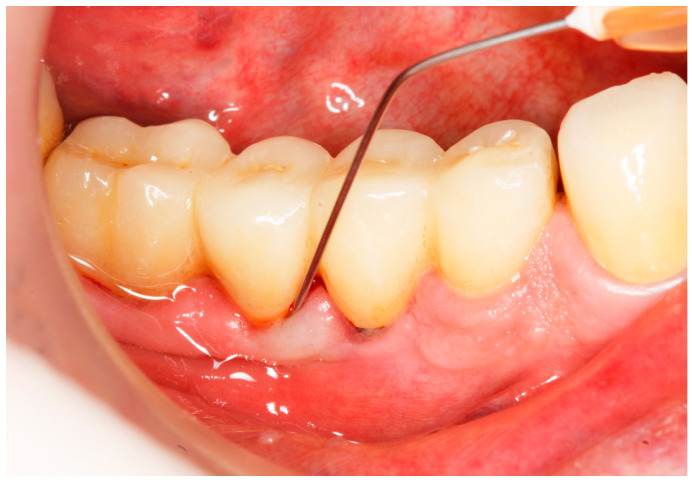
Application of Gelcide^®^ in the peri-implant sulcus.

Baseline: Prior to PiM treatment, PMPR was performed in all subjects (Figure 3), for both teeth and implants. Each patient was assigned to one of the two treatment groups according to computer-generated randomization. One implant (the one with the greatest number of BoP points or the presence of a line of bleeding or profuse bleeding) with PiM in each patient was selected for the study. PMPR of the implants of interest was performed using an ultrasonic scaler with a plastic tip (Piezon^®^ Master700 with Piezon^®^ PI instrument, EMS, Nyon, Switzerland), and air polishing (Perioflow^®^ handpiece, Airflow^®^ PLUS powder, EMS, Nyon, Switzerland) was carried out at all sites. Piperacillin plus tazobactam gel Gelcide^®^ (Italmed, Firenze, Italy) was prepared according to the manufacturer’s recommendations and was applied as follows: the solution was injected into the powder container, and the container was shaken until the solution became homogeneous. After mixing, the consistency of the mixture slightly increased, so it could be applied at the implant surface. Instrumentation was followed by subgingival application of the volatile mixture, depending on the patient’s group. For the Gelcide^®^ group (test), topical application of Gelcide^®^ was performed for the placebo group (control), topical application of a placebo (the volatile polymeric carrier alone) was carried out. The necessary quantity (not standardized) was then extracted from the mixed container with a syringe and inserted into the peri-implant sulcus at the apical extremity (Figure 4) until the excess became visible at the mucosal margin. Once the product had been applied, the excess was removed using a cotton ball.

**Figure 5:**
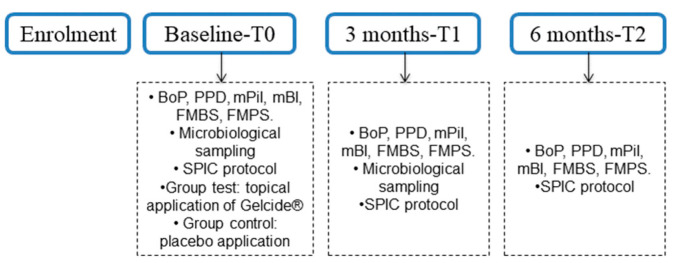
The timeline of the treatment. Abbreviations: BoP: bleeding on probing; PPD: implant probing pocket depth; mPil: modified plaque index; mBI: modified bleeding index; FMBS: full-mouth bleeding score; FMPS: full-mouth plaque score; SPIC: supportive peri-implant care protocol.

The post-application instructions for both groups were as follows: gentle brushing of the application area performed twice a day, removal of interdental plaque once a day, and rinsing with 0.20% chlorhexidine solution (Dentaton^®^, Ghimas s.p.a., Casalecchio di Reno, Italy) twice a day for two weeks following treatment. All participants received standardized oral hygiene instructions on using manual or power-driven toothbrushes and interdental brushes. The timeline of the treatment is represented in Figure 5.

### 2.9. Evaluation of Treatment Effect

**Figure 6:**
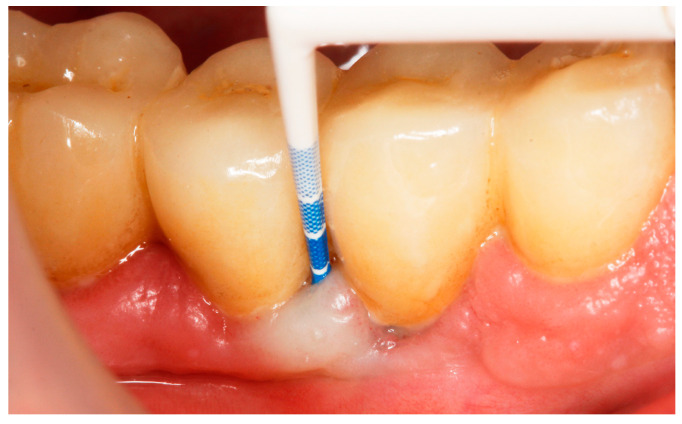
Pocket probing depth of 3 mm without BoP after 6 months.

Follow-up visits and reevaluations took place 3 and 6 months (Figure 6) after the initial evaluation. At 3 and 6 months, clinical parameters were re-measured. Microbiological samples were collected at baseline and 3 months. The SPIC protocol was applied at each recall. Participants received conventional professional prophylaxis and air polishing at each reevaluation (3 and 6 months). Oral hygiene instructions were reinforced if necessary. At the first reevaluation, it was checked adverse effects or concomitant use of medication that was against the inclusion criteria were present.

### 2.10. Rescue Protocol

The end point of PiM treatment at implant level was considered to be the existence of ≤1 point of BoP and an absence of suppuration. If, during the study, at reevaluation, a change in PPD around the peri-implant tissues showed an increase of ≥2 mm when compared with the previous measurement, and/or overt suppuration was present, appropriate treatment for PI was performed and the patient was excluded from the study.

### 2.11. Statistical Analysis

**Sample size calculation:** In the absence of reliable data on changes in clinical measures with regard to the use of a single dose of piperacillin plus tazobactam gel following non-surgical mechanical debridement, we assumed a mean difference of one BoP-positive site (of six sites per implant), a standard deviation of 1.00 between groups, a 74% power, and a significance level of 0.05. This resulted in a sample size estimation of 15 individuals in each group. 

**Study outcomes:** The primary outcome variable was the change in the number of peri-implant sites with BoP (baseline—6 months), while PPD, mPlI, mBI, FMPS, FMBS, and the detection scores of the changes in the five selected bacterial species (*A. a.*, *P. g.*, *T. f.*, *P. i.* and *T. d. A. actinomycetemcomitans*, *P. gingivalis*, *P. intermedia*, *T. forsythia* and *T. denticola* were regarded as secondary outcomes. For clinical outcomes, mean values and standard deviations (mean; SDs) of clinical parameters for all sites and buccal, lingual/palatal, and proximal sites were calculated per patient. The patient was the statistical unit. Changes between baseline and 3 months, baseline to 6 months, and 3 to 6 months visits were calculated. 

**Statistical Analysis**: Each quantitative variable (BoP, PPD, mPlI, mBI, FMPS, FMBS) was averaged for each patient at each time point and then utilized in the statistical analysis. Analyses were conducted at the patient level. Mean values and standard deviations (mean; SD) for the clinical parameters were computed for both groups. The disparities between the test and control groups for characteristics measured on a continuous or ordinal scale were assessed using the Mann–Whitney U test, as required. Proportions were analyzed using the chi-square test. Intragroup differences between successive time points for quantitative variables were analyzed using the Friedman test, followed by the Wilcoxon signed-rank tests for pairwise comparisons. The Bonferroni correction was employed to adjust for multiple comparisons. *p* values less than 0.05 were considered statistically significant. The statistical analyses were conducted using R software version 4.1.2 [51,52]. The detection frequency scores of the principal keystone bacteria were assessed in relation to the microbiological status. The results were noted and classified into one of four categories: 0 = nondetectable; 1 = detectable < 10^4^ (10^3^ for *A. actinomycetemcomitans*); 2 = 10^4^–10^5^ (10^3^–10^4^ for *A. actinomycetemcomitans*); 3 = 10^5^–10^6^ (10^4^–10^5^ for *A. actinomycetemcomitans*); and 4 ≥ 10^7^ (10^6^ for *A. actinomycetemcomitans*) [49]. Using the Wilcoxon signed rank test, intragroup comparisons of the detection scores of pathogen species between the baseline and 3-month reevaluation time points were made. For intergroup comparisons of the detection scores at each time point, the Mann–Whitney U test was applied.

## 3. Results

### 3.1. Subject Accountability

**Figure 7:**
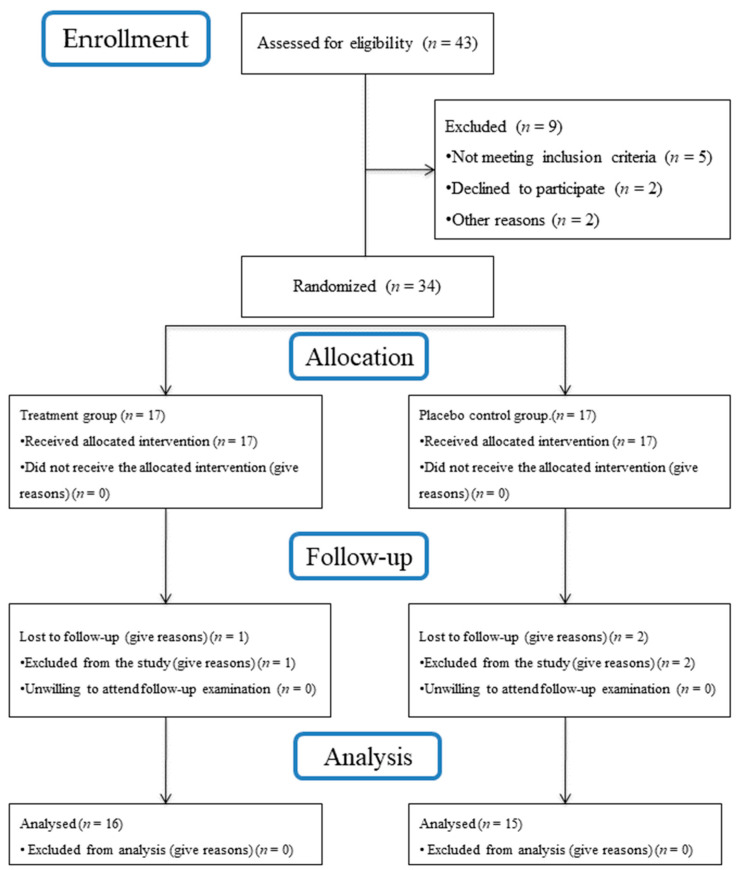
Flow chart of study procedures.

Figure 7 summarizes the flow chart of the study. Forty-three subjects were assessed for their eligibility. Five subjects did not meet the inclusion criteria, while four declined to participate. Consequently, a total of 34 patients with 34 implants (18 females and 16 males) were enrolled in the study and randomly assigned to the test or control procedures. Three patients, one from the test group and two from the control, were excluded from the study due to antibiotics prescribed during the follow-up for non-dental-related infections; they did not attend the 6-month follow-up examination and, therefore, were excluded from the final analysis. Therefore, thirty-one subjects (16 test and 15 control) received the allocated procedures and were included in the statistical analysis. 

### 3.2. Study Participants Characteristics 

The baseline characteristics of the 31 participants attending the 6-month follow-up are displayed in Table 1. The mean ages of the participants were 48.18 ± 5.41 and 50.46 ± 7.92 years for the test and control groups, respectively. Two patients in the test group and one in the control group were current smokers (fewer than 10 cigarettes per day). No adverse effects were recorded following the administration of the product test and the placebo, or were reported by any patient during the whole observation period.

With respect to the implant position (i.e., maxilla anterior vs. mandible anterior or maxilla posterior vs. mandible posterior), none of these parameters showed statistically significant differences between the groups (*p* > 0.05). A similar percentage of implants had been placed in posterior, as well as in anterior areas of mandible and maxilla in both groups. The patient characteristics at baseline were not significantly different (*p* > 0.05) between the groups (Table 1). No rescue treatment was necessary at any follow-up visit.

### 3.3. Clinical Outcomes

#### 3.3.1. Bleeding on Probing

At baseline, the mean number of sites per implant presenting positive BoP was slightly greater in the test group, but the difference was not statistically significant. Table 2 summarizes the changes in the number of BoP-positive implants from baseline to 3 months and 6 months following therapy in the test and control groups, respectively. Both groups showed a gradual decrease in the number of sites with BoP. After 6 months, the number of implants with BoP decreased statistically significantly from baseline to 3 months and from baseline to 6 months (*p* < 0.001) in the test group and the control group, respectively. Significant differences (*p* = 0.039) were observed between groups at 6 months (difference to baseline) following therapy, in favor of the test group.

#### 3.3.2. Pocket Probing Depth

Table 1 presents the number and frequency distribution of sites with different baseline PPDs in the test and control groups, and shows the number and prevalence of the sites that received the treatment. The PPDs of the sites ranged from 2 to 5 mm at baseline, and the mean value of 6 sites per implant was calculated at baseline and at 3 and 6 months. PPD measurements demonstrated no significant differences between the study groups at baseline. Table 2 presents the changes in PPD from baseline to 3 and 6 months following therapy in the test and control groups, respectively. After 6 months, the mean PPD decreased in both groups, from 3.30 ± 0.48 mm to 2.92 ± 0.28 mm (test) and from 3.21 ± 0.38 mm to 3.00 ± 0.33 mm (control), respectively, but no statistical significance was achieved (*p* = 0.56). No statistically significant differences (*p* > 0.05) were observed with respect to PPD between groups after 3 months or at the 6-month follow-up. 

#### 3.3.3. Plaque Index

As shown in Table 2, the values of mPiI at implant level did not change significantly at 3 months (T1) or 6 months (T2) in either the test or the control group (T1: 0.26 ± 0.18; 0.32 ± 0.16; T2: 0.39 ± 0.21; 0.38 ± 0.17) (*p* > 0.05). No significant differences were observed between the control group and the test group at any time point or over time. The mean mBI changes at the implant level in both the test and control groups showed statistically significant reductions (*p* < 0.001) compared with the baseline. The intergroup comparison showed a statistically significant reduction in mBI between groups (*p* > 0.05, at 3 and 6 months of follow-up, respectively) in favor of the test group.

#### 3.3.4. Full-Mouth Plaque Score and Full-Mouth Bleeding Score

The full-mouth plaque score (FMPS) and full-mouth bleeding score (FMBS) at baseline and at the 3- and 6-month follow-ups are summarized in Table 2. At 3 and 6 months following therapy, the mean FMPS scores revealed statistically significant changes compared with the baseline in both groups. 

#### 3.3.5. Treatment Success (BoP ≤ 1 Site) at 6 Months following Therapy

The number and percentage of implants where the presence of a single bleeding spot around the implant was achieved is summarized in Table 3. The Chi-square statistic was 0.8186, and the *p*-value was 0.366. The result was not significant at *p* < 0.05. Therefore, there was no statistical difference between the number of sites with a single BoP ≤ or >1 after treatment.

### 3.4. Microbiological Results

The results of the microbiological analysis are presented in Table 4. No statistically significant differences were noted in the assessment of *A. actinomycetemcomitans*, *P. gingivalis*, *P. intermedia*, *T. forsythia*, or *T. denticola* between groups either at baseline or at 3 months of follow-up. The detection scores in the intergroup analysis decreased; however, the differences between the groups were not statistically significant, which is not surprising, since peri-implantitis was not present at baseline.

## 4. Discussion

Several studies have shown that anti-infective treatment protocols and the use of local antibiotics, among the more popular adjunctive treatments, do not lead to a complete resolution of mucosal inflammation around implants [53,54,55,56,57,58]. The aim of the present RCT with a 6-month duration was to evaluate the impact of a single application of piperacillin plus tazobactam gel, which was used as an adjunct to PMPR in PiM lesions. To our knowledge, no scientific data have yet validated the effectiveness of Gelcide^®^ (piperacillin plus tazobactam) in the treatment of PiM. The outcomes in our study failed to detect any statistically significant differences in clinical or microbiological outcomes after six months of follow-up. The only statistically significant difference was obtained in BoP reductions in the test group (differences to baseline), with significantly higher reductions when compared to the placebo group (*p* = 0.039).

In this study, the recent recommendations of the ID-COSM initiative [9], in particular the recommendations pertaining to the outcome measures employed in clinical studies on PiMs, were followed. The mandatory outcome domains in all trials include the evaluation of (i) surgical morbidity and complications until the final delivery of the prosthesis; (ii) the health status of the peri-implant tissue; (iii) adverse events related to the intervention; (iv) complication-free survival; and (v) the overall satisfaction and comfort of the patient [9]. The efficiency of the SPIC protocol for the management of PiM, as well as the outcomes, were evaluated at 3 months, in line with the recommendations made by the S3-level CPG, and if relevant end points had not been achieved, re-treatment was recommended [6].

The follow-up period of 6 months was not sufficient to offer enough data to provide precise results on effective prevention. The strict plaque control in both treatment groups and the PMPR provided at baseline and at three and six months led to improvements in both groups. This may have shadowed the possible differential impact of the adjunctive chemical therapy used in the test group, since a recent systematic review [59] demonstrated the importance of strict plaque control and maintenance protocols. The results of the present study over six months demonstrated similarly good improvements in the plaque scores at the treated implants. This reduction can be related to systematically reinforcing plaque control during the study, and not to a single application of the adjunctive agent, which apparently did not influence the plaque at the implant sites.

The strict plaque control in both treatment groups and the PMPR provided at baseline and at three and six months, may have shadowed the possible differential impact of the adjunctive chemical therapy used in the test group, since a recent systematic review [59] demonstrated the importance of strict plaque control and maintenance protocols in preventing PI. The results of the present study over six months demonstrated similarly good improvements in the plaque scores at the treated implants for both study groups and for the FMPS. This reduction can be related to systematically reinforcing plaque control during the study.

Bleeding on gentle probing is currently recognized as the key parameter for the diagnosis of PiM [2] because of its association with inflammation of the mucosa at the histological level [60]. Therefore, the clinical endpoint following non-surgical treatment of PiM is considered as the “concomitant absence of BoP (≤1 spot/implant), SOP, shallow PPD (≤5 mm) and absence of MBL loss” [9]. In our study, at the 6-month follow-up, this endpoint was achieved in 56.25% and 40% of implants (BoP values; the other requirements had already been fulfilled at baseline and remained stable over the follow-up period) in the test and control groups, respectively. Here, the slightly better values in the control group can be explained by the higher mean BoP values at baseline in the test group. Nevertheless, these findings are in line with those of other clinical studies evaluating the treatment of PiM lesions with mechanical instrumentation alone [5], in combination with the topical application of chlorhexidine [17,18,32], or with glycine powder [61,62]. Comparable disease resolution was recently obtained following non-surgical mechanical therapy of naturally occurring PiM in 43.6% tissue-level and 40% bone-level implants after non-surgical mechanical debridement [63]. A recent systematic review reported post-treatment BoP scores between 14.7% and 47.5% in PiMs, and no non-surgical protocols tested resulted in the complete resolution of inflammation at all implant sites [18]. Despite an improvement in clinical parameters in all sites and a statistically significant reduction in BoP, the resolution of PiM was not achieved in all patients in our study, and, furthermore, was not better in the test group. Other studies have evaluated the effects of other local antimicrobials formulas. In a 3- to 6-month follow-up period, adjunctive use of the local antimicrobials led to similar changes in BoP scores [28,31,64] and PD values [25,28,64] compared to control treatments (i.e., mechanical debridement alone), whereas one study reported a greater PD reduction following the adjunctive use of local CHX (0.12%) applications [31].

Comparable treatment outcomes with our study were obtained in one RCT following non-surgical mechanical therapy for PiM (8 patients, 24 implants, with clinical signs of PiM defined as: PD > 4 mm; BoP+; no radiographic bone loss) after using local delivery of tetracycline HCl (25%) fibers for 10 days (test) or mechanical debridement alone (control). This was the only RCT using locally delivered antibiotics to which we could refer. With the presence of this strong local antibiotic treatment, the test group revealed some BoP reduction; meanwhile, the control group showed increased BoP at 3 months [26]. The results of the present study demonstrated statistically significant differences between groups only for BoP. Potential reasons for this could be the single application of the product or the reported limited reliability of periodontal probing in evaluating peri-implant health [65], as these measurements can be significantly influenced by factors such as gender [66], probing force [67], and inadequate access to insert the periodontal probe [68]. Also, in contrast to periodontal tissues, the response of peri-implant tissue is influenced by various factors apart from the accumulation of biofilm [10], like the presence of residual cement, lack of keratinized mucosa, and the type of abutment material or prosthetic used, which can hinder oral hygiene control [15,69]. These factors may contribute to the differences in the results, as there are greater variations in the measured parameters compared to gingivitis. This makes it more challenging to assign the detection of significant therapeutically benefits to the use of locally delivered adjunctive antibiotic agents in managing PiM [32].

The reversibility of PiM is not necessarily reflected by the PPD reduction during the treatment [64], but the authors observed a weak correlation between PPD and BoP at mucositis sites. The PPD changes in the current study are in accordance with data from previous trials on mechanical treatment combined with anti-infective decontamination, which reported PPD reductions of 0.40 to 0.63 mm [17,25,61], while in our study, PPD reductions were obtained after 3 and 6 months between the test (0.38 ± 0.26 mm) and control (0.21 ± 0.20 mm) groups, respectively, and there was no powerful effect of the adjunctive agent. To compare these findings with treatments using standard local antimicrobial adjunctive products, a relevant previous study reported no statistically significant differences in mean PPD reductions between the test and control groups at 1 or 3 months after chlorhexidine gel application [17].

Although the efficacy of the combination of piperacillin and tazobactam against bacteria associated with periodontitis is unclear, the occurrence of β-lactamase-positive subgingival bacterial species in more than the half of the subjects with severe chronic periodontitis raises questions about the therapeutic potential of single-drug regimens with β-lactam antibiotics, like piperacillin, in periodontal therapy [70]. The results of this study indicate that the microbiological benefits of piperacillin plus tazobactam are comparable with those mentioned in another study that used local minocycline microspheres as an adjunct to mechanical debridement in the treatment of incipient PiM. At 6 months, no statistical significance was obtained between the groups for any bacteria or at any time point [29]. Our patients showed an overall low prevalence of detection of *P. gingivalis*, *P. intermedia*, *T. forsythia*, *A. actinomycetemcomitans*, and *T. denticola.* An explanation can be the fact that the tip of the paper point which was used herein may have only collected a small portion of the bacterial flora present in the peri-implant sulcus, which would increase the chance of obtaining false negative samples when compared to a selective culture method [29].

The variety of different implant types, diameters, surfaces, and topographies was not a variable of the study protocol; in our study, 90% of the analyzed implants were of the same type (AnyRidge, Megagen Implant Co. Ltd., Daegu, Republic of Korea), and the vast majority of them were 4 mm in diameter. These variables may complicate a generalization of the present study results, but based on previous clinical trials in PiM, “no relationship could be found regarding peri-implant probing depth (*p* = 0.456), type of implants connection (*p* = 0.623), nor implant position in the arch (*p* = 0.740)” [71]. Furthermore, “Implant diameter had no significant effect on peri-implant crestal bone loss (*p* = 0.098)” [72].

The present study can contribute to pursuing evidence-based recommendation therapy that aims to reduce soft tissue inflammation by using locally administered antibiotics as adjuncts, which may change as new evidence emerges. The limitations of this study include the unknown duration of the effect of the adjunctive topical administration of the applied substance, the unknown duration of persistence of the product on the submucosal implant surface, and the relatively short follow-up period. The single application of the product is also a limitation of this study. Even though the effect decreased, the improvements seen during the follow-up period in the treatment of periodontitis with the same product may have been enough to suggest some benefit of the treatment in PiM, since LDDs are thought to have the strongest antimicrobial effects in the first few days after treatment, when they are still detectable in the subgingival sites [51]. A further important issue to consider is the evidence indicating that peri-implant tissues have a greater inflammatory response around the implant and a slower healing rate than the gingival inflammation around natural teeth [7,73,74]. Given the results, the necessity to evaluate the cost/efficiency issue with respect to the combination of the antibiotics piperacillin and tazobactam for local delivery in PiM should be also considered.

## 5. Conclusions

Within the limitations of the present study, the single use of a slow-release, locally applied antibiotic combination of piperacillin and tazobactam gel to PMPR led to a limited improvement in the clinical parameters of implants diagnosed with PiM. Statistically significant differences in BoP reduction occurred between the two study groups in favor of the test group, where *p* = 0.039 was found. No changes in other clinical or microbiological parameters were seen. No study group achieved a complete resolution of PiM (i.e., BoP ≤ 1 per implant).

## Figures and Tables

**Table 1:** Sociodemographic data, implant locations, and PPD distribution at baseline (T0).

	Total	Test Group	Control Group	*p*-Value
Number of patients	31	16	15	
Number of implants	31	16	15	
Age (years, mean ± SD)		48.18 ± 5.41	50.46 ± 7.92	0.538
Gender				0.365
Female (*n*, %)	16	7 (43.8%)	9 (60%)	
Male (*n*, %)	15	9 (56.2%)	6 (40%)	
Smoker				0.998
Smoker ≤ 10 cig/day, *n* (%)	3	2 (12.5%)	1 (6.67%)	
Never smokers, number (%)	28	14 (87.5%)	14 (93.33%)	
Implant position				0.238
Anterior maxilla	2	2	0	
Posterior maxilla	15	8	7	
Anterior ma ndible	2	1	1	
Posterior ma ndible	12	5	7	
PPD (*n*, %)		96	90	
2 mm		11 (11.45%)	13 (14.44%)	
3 mm		46 (47.91%)	48 (53.33%)	
4 mm		38 (39.58%)	26(28.88%)	
5 mm		1 (1.04%)	3 (3.33%)	

Abbreviations: PPD, implant probing pocket depth; cig, cigarettes; *n*, number; SD, standard deviation.

**Table 2:** Clinical variables at baseline (T0), 3 months (T1), and 6 months (T2).

Parameter	Group	N	Baseline (T0)		3-Month (T1)		Baseline–3 Months		*p*-Value Intra-Group	6-Month (T2)		Baseline–6 Months		*p*-Value Intra-Group
			Mean	SD	Mean	SD	Mean	SD		Mean	SD	Mean	SD	
**BoP**	Test	16	0.72	0.17	0.25	0.10	0.47	0.18	**<0.001**	0.28	0.10	0.44	0.18	**<0.001**
	Control	15	0.63	0.18	0.22	0.12	0.41	0.18	**<0.001**	0.31	0.16	0.32	0.20	**<0.001**
	*p*-value		0.094		0.471		0.199			**0.813**		**0.039**		
**PPD**	Test	16	3.30	0.48	2.89	0.27	0.41	0.33	**0.006**	2.92	0.28	0.38	0.26	**0.009**
	Control	15	3.21	0.38	3.00	0.33	0.21	0.22	0.116	3.00	0.33	0.21	0.20	0.125
	*p*-value		0.460		0.444		0.082			**0.560**		**0.060**		
**mPiI**	Test	16	0.55	0.41	0.26	0.18	0.29	0.36	**0.018**	0.39	0.21	0.15	0.33	0.300
	Control	15	0.57	0.42	0.32	0.16	0.25	0.35	0.106	0.38	0.17	0.18	0.33	0.294
	*p*-value		0.873		0.311		0.745			**0.851**		**0.967**		
**mBI**	Test	16	1.04	0.38	0.22	0.07	0.82	0.39	**<0.001**	0.28	0.10	0.76	0.40	**<0.001**
	Control	15	0.80	0.34	0.22	0.12	0.57	0.36	**<0.001**	0.31	0.16	0.48	0.38	**<0.001**
	*p*-value		0.068		0.981		**0.038**			0.813		**0.036**		
**FMPS**	Test	16	20.81	3.39	11.25	3.92	9.56	4.03	**<0.001**	14.62	2.80	6.18	2.90	**<0.001**
	Control	15	21.4	3.26	13.66	3.82	7.73	4.25	**<0.001**	16.66	2.41	4.73	3.26	**<0.001**
	*p*-value		0.719		0.083		0.232			**0.036**		0.232		
**FMBS**	Test	16	22.50	2.30	14.12	2.68	8.37	2.89	**<0.001**	16.81	1.60	5.68	2.86	**<0.001**
	Control	15	22.80	1.97	15.80	2.73	7	2.75	**<0.001**	18.26	2.68	4.53	2.53	**<0.001**
	*p*-value		0.809		0.135		0.201			0.082		0.105		

Abbreviations: PPD, probing depth; BoP, bleeding on probing; mBI, modified bleeding index; mPiI, modified plaque index; FMPS, full-mouth plaque score; FMBS, full-mouth bleeding score. SD, standard deviation. Notes, *p*-values in bold indicate statistically significant differences.

**Table 3:** Number and percentage of implants where the presence of a single bleeding spot around the implant was achieved.

	Test Group (*n* = 16) *n* (%)	Control Group (*n*= 15) *n* (%)	*p*-Value
BoP sites ≤ 1	9 (56.25%)	6 (40%)	0.366 *
BoP sites > 1	7 (43.75%)	9 (60%)	

* Chi-square test. Abbreviations: BoP—bleeding on probing.

**Table 4:** Detection scores for the species *A. actinomycetemcomitans*, *P. gingivalis*, *P. intermedia*, *T. forsythia*, and *T. denticola* at baseline and after 3 months in the groups (data are presented as frequencies %).

Species	Time Point	Detection Score	Test Group (*n* = 16)	Control Group (*n* = 15)	*p*-Value
*A. actinomycetemcomitans*	baseline	0	14 (87.50%)	15 (100%)	0.163
		1	-	-	
		2	1 (6.25%)	-	
		3	-	-	
		4	1 (6.25%)	-	
	3 months	0	14 (87.50%)	15 (100%)	0.163
		1	-	-	
		2	1 (6.25%)	-	
		3	-	-	
		4	1 (6.25%)	-	
	*p*-value		1	-	
*P. gingivalis*	baseline	0	3 (18.8%)	2 (25%)	0.779
		1	1 (6.25%)	1 (13.3%)	
		2	8 (50%)	8 (53.3%)	
		3	4 (25%)	4 (26.7%)	
		4	-	-	
	3 months	0	4 (25%)	2 (13.3%)	0.715
		1	2 (1.25%)	4 (26.7%)	
		2	9 (56.2%)	7 (46.7%)	
		3	1 (6.25%)	2 (13.3%)	
		4	-	-	
	*p*-value		0.240	0.263	
*P. intermedia*	baseline	0	10 (62.5%)	9 (60%)	0.783
		1	2 (12.5%)	-	
		2	3 (18.8%)	6 (40%)	
		3	1 (6.25%)	-	
		4	-	-	
	3 months	0	13 (81.2%)	12 (80%)	0.931
		1	1 (6.25%)	1 (6.67%)	
		2	2 (12.5%)	2 (13.3%)	
		3	-	-	
		4	-	-	
	*p*-value		0.242	0.194	
*T. forsythia*	baseline	0	2 (12.5%)	1 (6.67%)	0.796
		1	1 (6.25%)	1 (6.67%)	
		2	6 (37.5%)	8 (53.3%)	
		3	7 (43.8%)	5 (33.3%)	
		4	-	-	
	3 months	0	5 (31.2%)	1 (6.67%)	0.535
		1	2 (12.5%)	4 (26.7%)	
		2	5 (31.2%)	7 (46.7%)	
		3	4 (25%)	3 (20%)	
		4	-	-	
	*p*-value		0.138	0.242	
*T. denticola*	baseline	0	4 (25%)	3 (20%)	0.074
		1	8 (50%)	2 (13.3%)	
		2	4 (25%)	10 (66.7%)	
		3	-	-	
		4	-	-	
	3 months	0	8 (50%)	10 (66.7%)	0.446
		1	6 (37.5%)	3 (20%)	
		2	2 (12.5%)	2 (13.3%)	
		3	-	-	
		4	-	-	
	*p*-value		0.148	**0.003**	

*p*-values in bold indicate statistically significant differences.

## Data Availability

The data presented in this study are available upon request from the corresponding author.
